# Chemical Profile of the Sulphated Saponins from the Starfish *Luidia senegalensis* Collected as by-Catch Fauna in Brazilian Coast

**DOI:** 10.1007/s13659-018-0153-2

**Published:** 2018-01-22

**Authors:** Marcelo Marucci Pereira Tangerina, Júlia Pizarro Cesário, Gerson Rodrigues Raggi Pereira, Tania Márcia Costa, Wagner Cotroni Valenti, Wagner Vilegas

**Affiliations:** 1 UNESP- Univ Estadual Paulista, Coastal campus of São Vicente, Praça Infante Dom Henrique s/n, São Vicente, SP 11330-900 Brazil; 2 Chemistry Institute, São Paulo State University – UNESP, Araraquara Campus, Prof. Francisco Degni, 55, SP, Araraquara 14800-060 Brazil

**Keywords:** *Luidia* senegalensis, By-catch fauna, Asterosaponins, UPLC-MS

## Abstract

**Abstract:**

The by-catch fauna of the shrimp fishery includes a number of marine invertebrates that are discarded because they do not have commercial value. In order to try to add some value to these materials, we analyzed the chemical composition of the starfish *Luidia senegalensis* collected in the Brazilian coast as a consequence of the trawling fishery method. In order to access their chemical composition, we used a combination of solid phase extraction (SPE) followed by ultra-high performance liquid chromatography coupled to electrospray ionization ion trap tandem mass spectrometry (UPLC-ESI-IT-MS^n^). *Luidia senegalensis* contains asterosaponins, which are sulphated glycosilated steroids, containing five and six sugar moieties, in addition to polyhydroxysteroids. This study helped us to support the presence of important and potentially bioactive compounds in invertebrates associated to the by-catch fauna of the shrimp fishery, using a fast and efficient method.

**Graphical Abstract:**

## Introduction

Waste of resources from biodiversity is huge in Brazil and worldwide. The broad variety of marine organisms (algae, mollusks, sponges, corals, etc.) that are caught together with fish and shrimp is completely ignored as a source of new molecules. Due to the low economic value, the fishermen despise them, because they are not marketable as food. The shrimp fishery is one of the most striking marine ecosystems: the use of systems of seabed trawling causes the destruction of all region where it is employed, capturing up to 21 kg of by-catch fauna kg^−1^ of shrimp [1, 2]. However, several substances found in marine organisms can be widely applied in many areas to improve the quality of life of humanity, including the areas of food, power generation and medicine. Therefore, we investigate the chemical compounds present in the by-catch fauna of the shrimp fishery on the coast of State São Paulo, Brazil, in order to obtain molecules of potential economic interest, which can increase the value of this wasted material. To start our work on the chemical composition of the by-catch fauna of the shrimp fishery, we have investigated the polar extract of the nine-armed starfish *Luidia senegalensis* Lamark collected in the coast of the São Paulo State, Brazil. *Luidia* Forbes (1839, Luidiidae, Asteroidea: Paxillosida) are bottom sea stars, which live in sandy or muddy substrate. The genus includes 49 species that occur in tropical and subtropical shallow waters [3]. *Luidia senegalensis* [syn. *Asterias senegalensis* Lamarck (1816), *Luidia marcgravii* Steenstrup in Lutken (1859, synonym according to Perrier (1875)] occurs at depths of up to 40 meters alongside the coast of South America, including southern Brazil, as well as around the coasts of Florida, in the Caribbean Sea and the Gulf of Mexico [4].

Starfish (called also sea stars) are marine invertebrates widely recognized as an outstanding source of natural products. They belong to the class Asteroidea, phylum Echinodermata. The chemical composition of a number of starfishes has been investigated using several chromatographic and spectrometric techniques. This class of Echinodermata is rich in free polyhydroxysteroids and two main groups of steroid glycosides: asterosaponins and the glycosides derived from the polyhydroxysteroids. The asterosaponins present a ∆^9,11^−3β,6α-steroidal core, with four rings, a sulphate group at C3, one or two oxygenated carbons at the side chain, and four to six sugar moieties attached to C-6. Common saccharide residues are pentoses (xylose, arabinose), deoxyhexoses (quinovose, fucose), hexoses (glucose, galactose) and 6-deoxy-xylo-hex-4-ulose (DXHU). Therefore, the extracts produced from these marine organisms are often very complex mixtures of free and sulphated highly oxygenated compounds as well as their sodium salts. These sulphated steroid oligoglycosides usually have molecular weight higher than 1200 Da and may include isomeric compounds. These substances have a wide variety of pharmacological activities. Among them, they act as anti-viral, anti-bacterial, anti-inflammatory, antifungal, hemolytic, activate tubulin polymerization, inhibit tumour proliferation and possess immunomodulatory activities. They are also involved in physiological and chemical defense, interspecific chemical communication, digestion and reproduction [5, 6]. Recently, mass spectrometry coupled or not to liquid chromatography has been proved to be a powerful tool for the investigation of the saponins present in the polar extracts of sea stars, without the need of prior separation [7, 8].

In our case, the analysis of the polar hydroethanolic extract of *L. senegalensis* was accomplished using a combination of solid-phase extraction (SPE), direct flow injection-electrospray-ion trap tandem mass spectrometry (DFI-ESI-IT-MS^n^) and an ultra-high performance liquid chromatography-electrospray-ion trap tandem mass spectrometry (UPLC-ESI-IT-MS^n^) method. Substances were tentatively identified using the typical fragmentation of the aglycone side chain, the characteristic losses of the sugar moieties and comparison with the fragmentation pattern described in the literature [6–10].

## Results and Discussion

In this study, we assessed the chemical profile of the saponins of the starfish *Luidia senegalensis* collected as by-catch fauna of the shrimp fishery in Brazil, as a result of trawling employed by traditional fishers, who use to discard these organisms. No chemical investigation of this marine invertebrate could be found in the literature. Starfish present many interesting compounds, which are usually derived from sulphated steroids, with a wide variety of substitutions in the aglycone core (e.g. hydroxylation pattern, side chain arquitecture) [6–10].

The putative structure of the saponins detected in *L. senegalensis* were associated mainly with those found in *Aphelasterias japonica* [7], *Asterias rubens* [8], *L. maculata* [9] and in *L. quinaria* [10], besides those described in the review published by Dong et al. [6]. In view of the fact that many of the starfish saponins contains similar molecular weight but different aglycone design and a number of oligosaccharide chains, the confirmation of the structures requires further chemical investigation using additional spectrometric techniques (e.g. Nuclear Magnetic Resonance, Circular Dichroism, etc.). Therefore, it is important to reinforce that the main goal of this work was not to fully identify the saponins, but instead to present an overview of the chemical composition of the discarded material considered as by-catch of the shrimp fishery.

### SPE Fractionation of the Crude Extract

The matrix used in these experiments is a mixture of inorganic salts (NaCl), organic salts (sulphated saponins), and amphoteric compounds (amino acids). To extract the secondary metabolites from the hydroethanolic crude extract and remove inorganic salts and amino acid impurities, we use a solid phase extraction in a C18 reversed phase (RP18 SPE) cartridge. After sequential elution with water/methanol gradient, thin layer chromatography (TLC) analyses in silica gel plates indicated that inorganic salts and amino acids remained only in the aqueous fraction (revealed as reddish spots when sprayed with ninhydrin reagent), whereas saponins appeared as purple spots in the hydromethanolic fraction (HF) [11]. Besides, UPLC-ESI-IT-MS analysis did not evidenced peaks corresponding to amino acids, which also shows that the SPE procedure used was good for the separation of the secondary metabolites from these starfishes, leading to the elimination of the amino acids.

### DFI-ESI-IT-MS^n^ Analyses

**Fig. 1:**
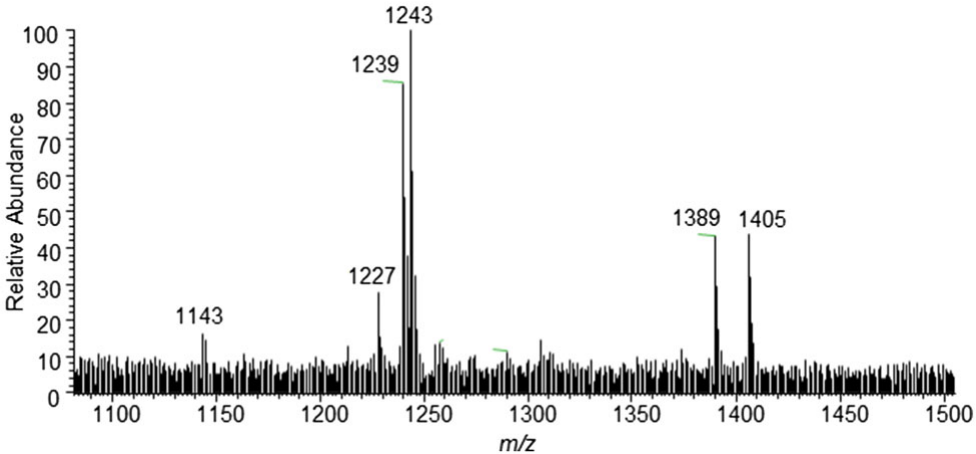
Full scan DFI-ESI-IT-MS^n^ (Negative Ionization) mass spectrum of *Luidia senegalensis* showing the peaks corresponding to the saponins

In order to obtain a preliminary fingerprint about the chemical composition of *L. senegalensis*, HF was directly injected into the ESI source of the ion trap. We have tested different mass spectrometry conditions and decided to use the negative ionization and optimized conditions as presented in the Experimental Section. This fact agrees with the literature, which reports intense [M–Na]^−^ signals in the range of *m/z* 1100–1400, corresponding to ionized saponins containing Na^+^–O_3_SO-groups [12 Demayer et al. [8] observed that, since the sulphate group is commonly attached to the position 3 of the aglycone core, the most intense signals of the mass spectra contains the aglycone nucleus. In our case, intense signals were observed in the *m/z* 1200–1500 Da range of the full scan mass spectra, that may be assigned to the presence of negatively ionized saponin ions [M–Na]^−^ (Fig. 1).

#### UPLC-ESI-IT-MS Analysis

**Fig. 2:**
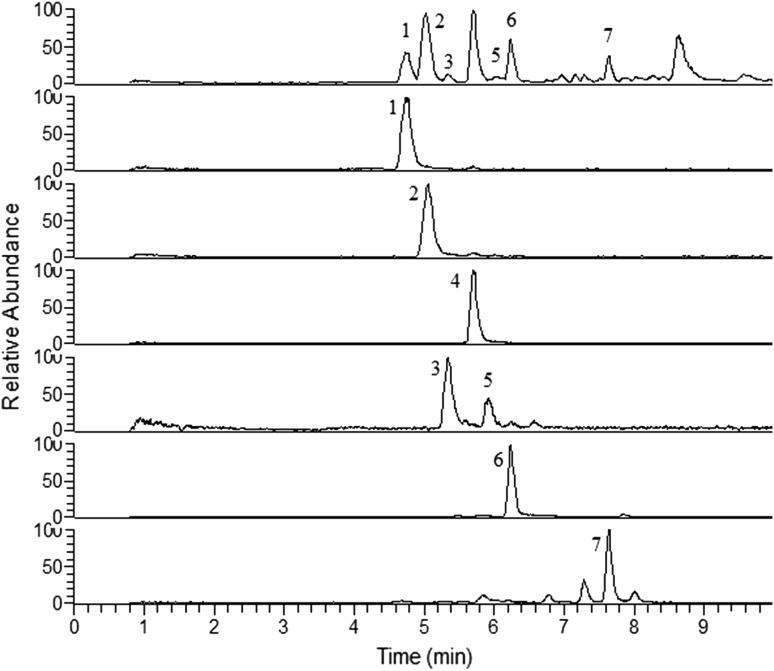
UPLC-ESI-IT-MS analysis of the saponins present in *Luidia senegalensis*. Base Peak Ion—BPI (above) and extracted chromatograms of the ions 1: *m/z* 1405; 2: *m/z* 1389; 4: *m/z* 1239; 3 and 5: 1227; 6: *m/z* 529; 7: *m/z* 547

Figure 2 displays the analysis of the HF from *L. senegalensis* by UPLC-ESI-IT-MS. After optimization of the solvent composition and elution gradient, the chromatogram showed a reasonable baseline separation for the peaks, which could be analyzed in a time interval of less than 10 min with little interference. It is worth mentioning that no baseline separation and strong peak tailing were observed when we performed chromatographic runs under the same conditions, but without acidifying the eluent mixtures with 0.1% formic acid.

**Fig. 3:**
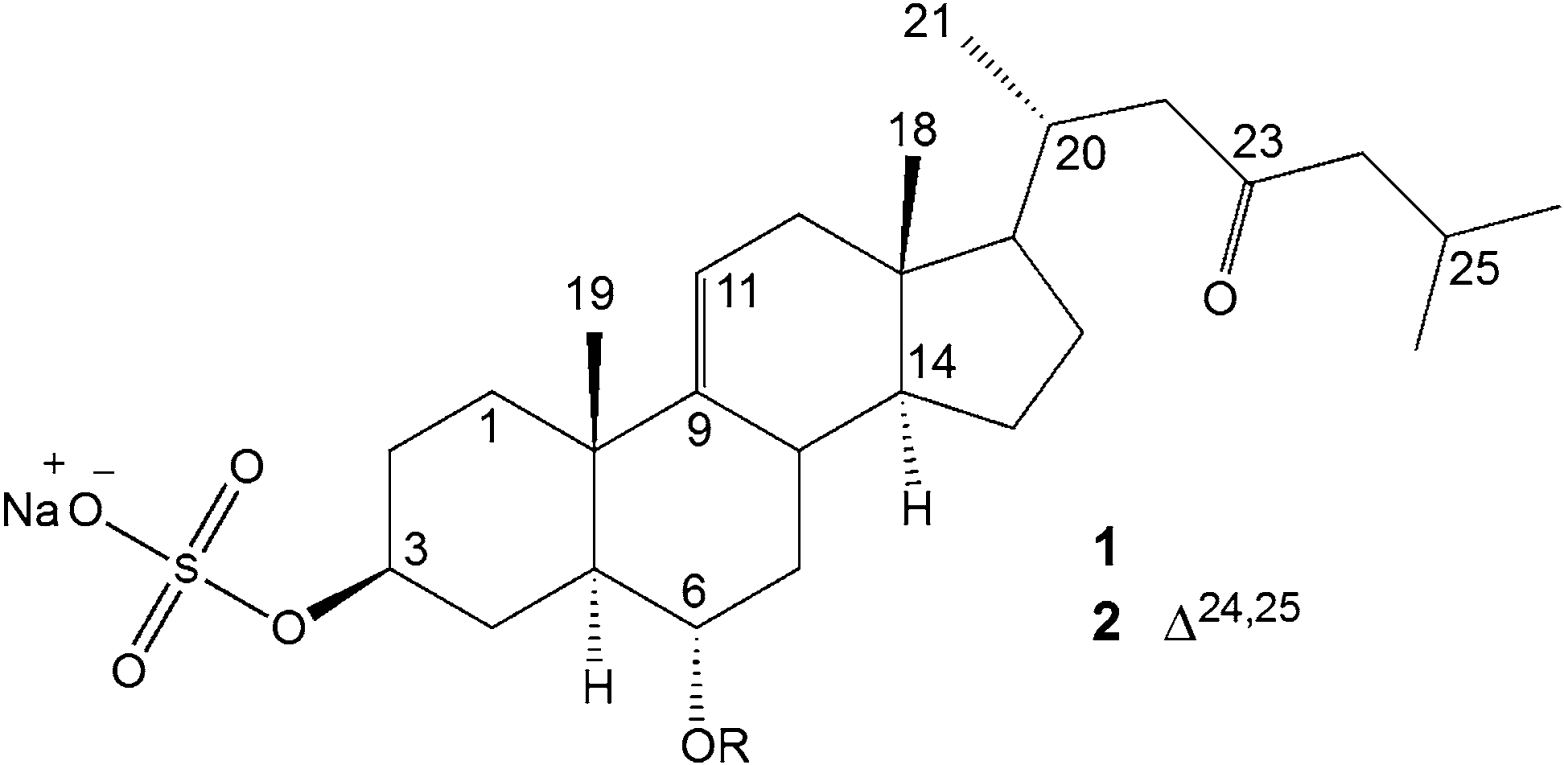
Aglycones of the steroidal saponins detected in *Luidia senegalensis*. R corresponds to the sugar sequence described in Table 1

Even using very strong source conditions (e.g. − 80 eV), UPLC-ESI-IT-MS experiments were not enough to produce extensive fragmentation of the precursor ions contained into the chromatographic peaks. Therefore, we performed UPLC-ESI-IT-MS^n^ experiments using the precursor ion of each chromatographic peak as well as the MS^2^, MS^3^ and MS^4^ product ions observed after the DFI-ESI-IT-MS^n^ experiments. Combination of DFI-ESI-IT-MS^n^, UPLC-ESI-IT-MS and UPLC-ESI-IT-MS^n^ led to the data displayed in Table 1, which shows the retention times, main fragment ions and the tentative assignment of the compounds. The putative identification is discussed ahead and the chemical structures of the compounds are shown in Fig. 3.

**Table 1:** Proposed saponins present in *Luidia senegalensis* detected by ESI-IT-MS^n(1)^, UPLC-ESI-IT-MS and UPLC-ESI-IT-MS^n(2)^ in negative ion mode

Peak	R_t_ (min)	Precursor íon (*m/z*)	Fragment ions (*m/z)*	MW	Tentative assignment	
					Aglycone	Sugar sequence
1	4.74	1405	1305^(1,2)^, 1243^(2)^, 1143^(1,2)^, 1097^(1,2)^, 997^(1,2)^, 935^(1,2)^, 835^(1,2)^, 789^(2)^, 657^(2)^, 557^(2)^	1428	1	Hex–dHex–Hex–Pent(–dHex)–Hex
2	5.06	1389	1289^(1,2)^, 1243^(2)^, 1143^(1,2)^, 1097^(1,2)^, 997^(1,2)^, 935^(2)^, 851^(1,2)^, 835^(1,2)^, 689^(1,2)^, 395^(2)^	1412	1	dHex–dHex–Hex–Pent(–dHex)–Hex
3	5.34	1227	1127^(1,2)^, 1081^(1,2)^, 981^(1,2^, 935^(1,2)^, 919^(1,2)^, 849^(2)^, 835^(1,2)^, 773^(1,2)^, 641^(1,2)^, 495^(1,2)^	1250	1	dHex–dHex–Pent(–dHex)–Hex
4	5.70	1239	1093^(2)^, 947^(2)^, 801^(2)^, 655^(2)^, 493^(2)^	1262	2	dHex–dHex–dHex(–dHex)–dHex
5	5.93	1227	1127^(1,2)^, 1081^(1,2)^, 981^(1,2^, 935^(1,2)^, 919^(1,2)^, 849^(2)^, 835^(1,2)^, 773^(1,2)^, 641^(1,2)^, 495^(1,2)^	1250	1	dHex–Hex–Pent(–dHex)–dHex
6	6.24	529	ND			
7	7.65	547	ND			

*ND* not determined, *dHex* deoxyhexose, *Hex* hexose, *Pent* pentose

In brief, the ions of [M–Na]^−^ were, in most cases, the major ones, and their main fragmentation pathway were neutral loss of sugar units. Typical losses of 132 Da, 146 and 162 Da were assigned to the presence of pentose [Pent, e.g., xylose (132)], deoxyhexose [dHex, e.g. fucose or quinovose (146)] and hexose [Hex, e.g. glucose, galactose or DXHU (162 Da)]. In some cases, we observed the simultaneous loss of two sugar units. Side chain fragmentation with 23-oxo substitution led to losses of 100 Da, due to the low energy McLaffery rearrangement of 6-member transition states, which generates the neutral molecule C_6_H_12_O (4-methylpent-1-en-2-ol) [5, 6]. These typical fragment ions associated with sugar and/or side chain losses were also observed in the MS^n^ spectrum of many sulphated saponins from starfish previously described in the literature [5–8].

**Fig. 4:**
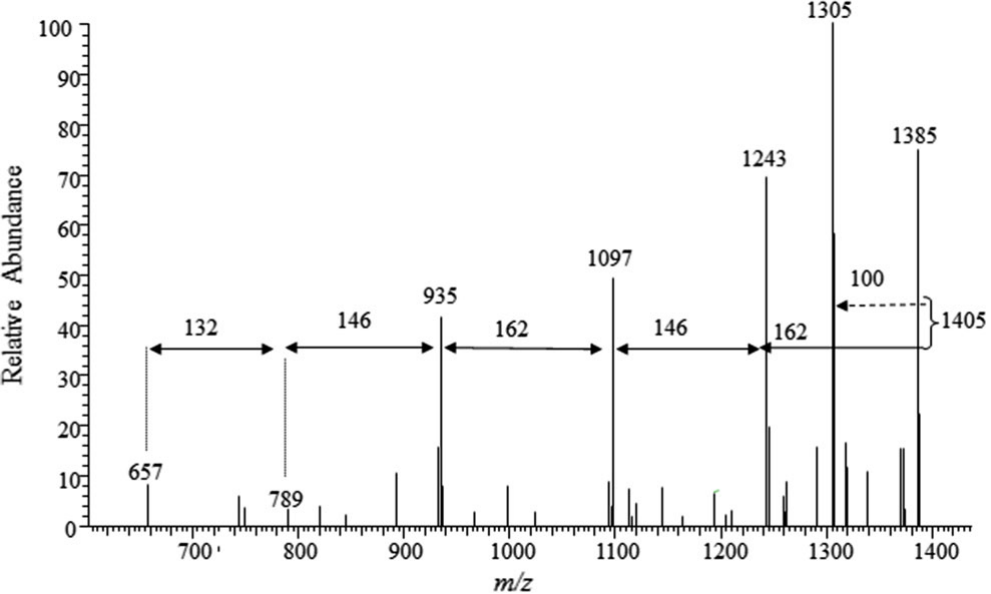
Mass spectrum obtained after UPLC-ESI-IT-MS^n^ experiment using the precursor ion

Using direct injection and CID dissociation, MS^2^ fragmentation of the precursor ion at *m/z* 1405 [M–Na]^−^ led to product ion at *m/z* 1305 [M–100-Na]^−^, assigned to the loss of the neutral molecule C_6_H_12_O, due to the McLafferty rearrangment. MS^3^ fragmentation of the parent ion at *m/z* 1305 gave the product ion at *m/z* 1143 [M-100-162-Na]^−^ (loss of a hexose unit). MS^4^ fragmentation of the precursor ion of *m/z* 1143 led to the product ion at *m/z* 997 [M-100-162-146-Na]^−^ (loss of a deoxyhexose). MS^5^ fragmentation of the precursor ion at *m/z* 997 led to the product ions at *m/z* 851 [M-100-162-146-162-Na]^−^ (loss of a hexose) and at *m/z* 835 [M-100-162-146-146-Na]^−^ (loss of a deoxyhexose), thus evidencing the presence of a branched saccharide chain. Due to the low abundance, further MS^n^ experiments were not practicable. Figure 4 displays an example of the UPLC-ESI-IT-MS^n^ analysis performed with the ion of *m/z* 1405 and the product ions up to MS^4^ (obtained from the DFI-ESI-IT-MS^n^ experiments), present in the peak with retention time 4.74 min. We could observe the production of several product ions, which corresponds to the fragmentation sequence 1405 → 1243 (loss of 162, hexose) → 1097 (loss of 146, deoxyhexose) → 935 (loss of 162, hexose) → 789 (loss of 146, deoxyhexose) → 657 (loss of 132, pentose) → 557 (loss of 100, C_6_H_12_O). Despite we could not observe the loss of the hexose attached to the aglycone, the combination of these data was consistent with the molecular formula C_62_H_101_O_33_SNa and allowed us to suggest the presence of the aglycone **1** attached to the sugar sequence Hex–dHex–Hex–Pent(–dHex)–Hex.

MS^2^ fragmentation of the precursor ion at *m/z* 1389 [M-Na]^−^ led to product ion at *m/z* 1289 [M-100-Na]^−^. MS^3^ fragmentation of the parent ion at *m/z* 1289 gave the product ion at *m/z* 1143 [M-100-146-Na]^−^ (loss of a deoxyhexose unit). MS^4^ fragmentation of the precursor ion of *m/z* 1143 led to the product ion at *m/z* 997 [M-100-146-146-Na]^−^ (loss of a deoxyhexose). MS^5^ fragmentation of the precursor ion at *m/z* 997 led to the product ions at *m/z* 851 [M-100-146-146-146-Na]^−^ (loss of a deoxyhexose) and at *m/z* 835 [M-100-146-146-162-Na]^−^ (loss of a hexose), again showing the presence of a branched saccharide chain. MS^6^ experiment of the parent ion at *m/z* 835 led to the product ion at *m/z* 689 [M-100-146-146-162-146-Na]^−^. The UPLC-ESI-IT-MS^n^ analysis of the peak with retention time 5.06 min produced the fragmentation sequence 1389 → 1243 (loss of 146, deoxyhexose) → 1097 (loss of 146, deoxyhexose) → 935 (loss of 162, hexose). These data are in agreement with the molecular formula C_62_H_101_O_32_SNa, which suggest the presence of aglycone **1** attached to the sugar sequence dHex–dHex–Hex–Pent(–dHex)–Hex.

MS^2^ fragmentation of the precursor ion at *m/z* 1227 [M-Na]^−^ led to product ions at *m/z* 1127 [M-100-Na]^−^ (loss of the side chain due to McLafferty rearrangement) and at *m/z* 1081 [M-146-Na]^−^ (loss of the terminal sugar moiety). Such co-occurrence of different fragmentation routes was already described in the literature and can be assigned to the presence of saponin isomers [5, 6]. Therefore, we first choose to proceed MS^3^ experiments with the fragmentation of the parent ion at *m/z* 1127, which gave the product ion at *m/z* 981 [M-100-146-Na]^−^ (loss of a deoxyhexose) and at *m/z* 1109 [M-100-18-Na]^−^ (loss of water). Demeyer et al. [6] demonstrated that the water loss is associated with the presence of a DXHU residue in the oligosaccharide chain. MS^4^ fragmentation of the precursor ion at *m/z* 981 gave the product ion at *m/z* 835 [M-100-146-146-Na]^−^. Due to the low abundance of the *m/z* 835 ion we could not perform further experiments. This fragmentation pattern and the molecular weight suggest molecular formula C_56_H_91_O_27_SNa, which prompted us to propose the presence of the aglycone **1** attached to the sugar sequence dHex–dHex–Pent(–dHex)–Hex. On the other hand, MS^3^ fragmentation of the precursor ion at *m/z* 1081 led to the product ions at *m/z* 935 [M-146-146-Na]^−^ (loss of a second deoxyhexose unit) and at *m/z* 919 [M-146-162-Na]^−^ (loss of an hexose), thus suggesting the presence of a branched saccharidic chain at C6. MS^4^ fragmentation using the parent ion at *m/z* 919 led to the product ion at *m/z* 773 [M-146-162-146-Na]^−^ (loss of a second deoxyhexose unit), at *m/z* 641 [M-146-162-146-132-Na]^−^ (loss of a pentose unit) and at *m/z* 495 [M-146-162-146-132-146-Na]^−^ (loss of a deoxyhexose). MS^4^ fragmentation of the ion at *m/z* 935 led to the same product ion at *m/z* 773 [M-146-146-162-Na]^−^ (loss of the hexose moiety). MS^5^ fragmentation of the precursor ion at *m/z* 773 led to the product ion at *m/z* 641 [M-146-146-162-132-Na]^−^ due to the loss of a pentose unit. MS^6^ fragmentation of the precursor ion at *m/z* 641 led to the product ion at *m/z* 495 [M-146-146-162-132-146-Na]^−^, consistent with the loss of a final deoxyhexose moiety. All these data suggest molecular formula C_56_H_91_O_27_SNa, and are compatible a saccharide sequence dHex–Hex–Pent(–dHex)–dHex linked to the aglycone **1**. These two molecules could account to the presence of two chromatographic peaks for the extracted ion of *m/z* 1227, at 5.34 and 5.93 min. MS^n^ fragmentation of the ions contained in these two chromatographic peaks did not led to good fragmentation, probably due to the low abundance, and were not considered.

MS^2^ fragmentation of the precursor ion at *m/z* 1239 [M–Na]^−^ led to product ion at *m/z* 1093 [M-146-Na]^−^, corresponding to the loss of a deoxyhexose unit. No fragmentation of the side chain was observed. Further MS^n^ fragmentation led to the sequential losses of four deoxyhexose moieties, producing the ions at *m/z* 947 [M-146-146-Na]^−^, at *m/z* 801 [M-146-146-146-Na]^−^, at *m/z* 655 [M-146-146-146-146-Na]^−^. In addition, fragmentation of the parent ion at *m/z* 655 led to the product ion at *m/z* 493 [M-146-146-146-146-162-Na]^−^, assigned to the loss of one hexose. The UPLC-ESI-IT-MS^n^ analysis of the peak with retention time 5.70 min produced the fragmentation sequence 1239 → 1093 (loss of 146, deoxyhexose) → 947 (loss of 146, deoxyhexose) → 801 (loss of 146, deoxyhexose) → 655 (loss of 146, deoxyhexose) → 493 (loss of 162, hexose). All these data are compatible with an asterosaponin with the formula C_55_H_87_O_27_SNa. Since the aglycone differs in 2 Da from aglycone **1**, we propose the presence of an additional double bond, like in compound **2**, attached to the sugar sequence dHex–dHex–dHex(–dHex)–Hex–Agycone. This proposal is similar to Ruberoside G, which was reported in the sea star *Asterias rubens* [6].

Peaks 6 and 7 did not present fragmentation in the UPLC-ESI-IT-MS experiments, and they might be due to the presence of polyhydroxysteroids, of large occurrence in starfish [4].

No evidence for either 20-hydroxy or 24-methyl side chain (loss of 114 Da due to a McLafferty rearrangment) was detected in our ESI-IT-MS^n^ experiments. Regarding UPLC analyses, separation of peaks containing structurally similar complex compounds bearing the same aglycone but different saccharide chain arrangements was easily achieved with a very short run time (e.g. hexasaccharide saponins: *m/z* 1405/1389; pentasaccharide saponins: *m/z* 1227).

## Conclusions

Our results demonstrate that noteworthy characteristics of *L. senegalensis* are the presence of highly polar asterosaponins with 5 and 6 sugar moieties. Datta et al. [3] described that sulphated steroidal saponins present several biological activities, like hemolytic, antineoplastic, cytotoxic, antitumor, antibacterial, antiviral, antifungal and anti-inflammatory. Besides, they could be involved in several spheres of living like chemical defense, digestion and reproduction [4]. As far as we are concerned, no previous reports on the chemical composition of this starfish was reported. Since that sugar identities and connectivity may vary in starfish saponins, it was not possible to fully identify these compounds in this study. On the other hand, we were not able to find in the literature compounds with the combination of aglycone and sugar sequences described in Table 1. Hence, despite asterosaponins are well-known secondary metabolites of starfish, it is possible that this marine invertebrate presents new molecules, which will be investigated in a near future. In the context of this study, the combination of direct injection ESI-IT-MS^n^ and UPLC-ESI-IT-MS^n^ experiments helped us to support the presence of important compounds in invertebrates associated to the by-catch fauna of the shrimp fishery, using a fast and efficient method. This result can contribute to a more rational and sustainable use of the Brazilian marine biodiversity resources.

## Experimental Section

### Chemicals and Materials

Methanol Chromasolv LC–MS-grade, chloroform, n-propanol, formic acid, ninhidrin, anysaldehyde and sulfuric acid were acquired from Sigma-Aldrich (São Paulo, Brazil). Ultrapure water was produced using a Milli-Q system (Millipore, Bedford, MA, USA).

### Animal Material, Extraction and Fractionation

Individuals of *Luidia senegalensis* were collected in the city of Ubatuba–SP, along with a shrimp fisherman by bottom trawling of 1 h, in April 19th, 2013, between the coordinates 2329′21″S/44°59′95″O and 23°33′01″S/45°00′54″O. The specimens were kept in ice for transportation and frozen after arriving in the laboratory until extraction. The starfish was identified by Prof. Dr. Tania Marcia Costa, from UNESP-Coastal campus of São Vicente. In addition, the specimens were photographed to afford a visual voucher.

The animals were thawed to room temperature, separated by species, crushed with mortar and pestles. One gram of each animal was extracted by maceration with 10 mL of ethanol 70% (3 days). The extract was filtered and the solvent was removed under vacuum at 40 °C, using a rotary evaporator.

The SPE cartridge (500 mg, Macherey–Nagel, Chromabond C18 ec, Düren, Germany) was first preconditioned by the consecutive passing of 5 mL of methanol and then 5 mL of pure water by gravity. The extract obtained was solubilized in water at concentration of 1 mg mL^−1^, filtered on filter paper to remove macro particles and further filtered through a 0.45 µm membrane of a nylon filter. The sample was loaded to the cartridge and first eluted with 5 mL of water (to eliminate salts and free amino acids). The cartridge was eluted again with 5 mL of water/methanol (90:10, v/v, named hydromethanolic fraction-HF) and finally with 5 mL of pure methanol. All three fractions were analyzed using two thin layer chromatography plates (TLC, silicagel, 20 × 20 cm, 250 µm layer, UV fluorescence 254 nm, Whatman Ltd, Maidstone, England; Eluent: chloroform:methanol:n-propanol:water 100:11:11:27 v/v) and separately revealed with ninhidrin (to detect amino acids) and anisaldehyde/sulfuric acid mixture (to detect secondary metabolites). Fraction collected in water contained only amino acids and other impurities and HF concentrated the compounds of interest. HF was transferred into clean tubes and dried under nitrogen gas at room temperature. The sample was redissolved in pure methanol/water 50:50, v/v to a concentration of 5 ppm and analyzed by mass spectrometry.

### ESI-MS^n^ Analysis

Direct flow infusion of the samples was performed on a Thermo Scientific LTQ XL linear ion trap analyzer equipped with an electrospray ionization (ESI) source, both in positive and negative modes (Thermo, San Jose, CA, USA). It was used a fused-silica capillary tube at 280 °C, spray voltage of 5.00 kV, capillary voltage of − 35 V, tube lens of − 100 V and a 5 μL min^−1^ flow. Full scan analysis was recorded in *m/z* range from 100 to 2000. Multiple-stage fragmentations (ESI-MS^n^) were performed using the collision-induced dissociation (CID) method against helium for ion activation. The first event was a full-scan mass spectrum to acquire data on ions in that *m/z* range. The second scan event was an MS/MS experiment performed by using a data-dependent scan on the desodiated molecules from the compounds of interest at a collision energy of 30% and an activation time of 30 ms. The product ions were then submitted to further fragmentation in the same conditions, until no more fragments were observed.

### UPLC-ESI-IT-MS Analysis

UPLC-ESI-IT-MS analysis were carried out in a Thermo Scientific ultra-performance liquid chromatography equipment, consisting of an Accela AS autosampler, a quaternary Accela pump 600 coupled with the LTQ XL mass spectrometer described above, operating under the same conditions. Chromatographic separations were performed on a non-polar column (Kinetex^®^ cor-shell, C18, 1.7 µm, 100 × 2.1 mm, Phenomenex, USA) at room temperature. The mobile phases consisted of eluent A (0.1% formic acid in water, v/v) and eluent B (0.1% formic acid in methanol, v/v). These eluents were delivered at a flow rate of 0.2 mL min^−1^ with a linear gradient program as follows: 40–100% B from 0 to 5.0 min. After maintaining 100% B for 5 min, the column was returned to its initial condition. Aliquots of 10 μL of the samples were injected into the UPLC-ESI-IT-MS system for analyses using the autosampler. In the UPLC-ESI-IT-MS^n^ experiments for each parent ion of *m/z* 1405, *m/z* 1389, *m/z* 1239 and *m/z* 1227 we used the four product ions obtained after MS^2^, MS^3^ and MS^4^ experiments and CID of 30 eV against each ion, using the same chromatographic conditions.
